# *TUBB3*/*STMN1*基因表达与非小细胞肺癌EGFR通路的相关性

**DOI:** 10.3779/j.issn.1009-3419.2013.10.09

**Published:** 2013-10-20

**Authors:** 波 王, 彬 王, 连斌 张, 向阳 初, 刚 余

**Affiliations:** 1 100853 北京，中国人民解放军总医院胸外科 Department of thoracic surgery, the General Hospital of People's LiberationArmy, Beijing 100853, China; 2 510663 广州，益善生物技术股份有限公司 SurExam Bio-Tech Co.Ltd., Guangzhou 510663, China

**Keywords:** 肺肿瘤, TUBB3表达, STMN1表达, EGFR通路, Lung neoplasms, TUBB3, STMN1, EGFR pathway

## Abstract

**背景与目的:**

已有研究表明分子标志物在指导肺癌个体化治疗中起着重要作用。本研究旨在探讨非小细胞肺癌（non-small cell lung cancer, NSCLC）抗微管类药物靶点TUBB3（tubulin, beta 3 class Ⅲ)/STMN1（stathmin 1）基因表达水平与EGFR（epidermal growth factor receptor）、KRAS（Kirsten rat sarcoma viral oncogene homolog）、BRAF（v-raf murine sarcoma viral oncogene homolog B）、PI3K（phosphatidylinositol-4, 5-bisphosphate 3-kinase）基因突变的相关性，为NSCLC预后及药物疗效判断提供参考依据。

**方法:**

回顾性分析我院经病理确诊的46例NSCLC患者手术切除的肿瘤标本，通过分支DNA-液相芯片法检测*TUBB3*、*STMN1*基因mRNA表达水平，通过xTAG液相芯片法检测*EGFR*、*KRAS*、*BRAF*、*PI3K*基因突变，对检测结果应用SPSS 19.0软件采用*Spearman*相关进行分析基因表达与基因突变的关系。

**结果:**

抗微管靶点TUBB3和STMN1存在很强的共表达性，*TUBB3*基因高表达时，*STMN1*趋向于高表达（*P*=0.006）。*EGFR* E21突变与TUBB3存在负相关关系：*EGFR* E21无突变时TUBB3趋向于高表达（*P*=0.004, 6）。*KRAS* E2突变与*STMN1*存在正相关关系：*KRAS* E2突变时STMN1趋向于高表达（*P*=0.038, 6）。

**结论:**

抗微管耐药相关因子TUBB3和STMN1与EGFR通路的关键因子突变相关，提示了*EGFR*突变和*KRAS*突变是调控TUBB3/STMN1表达的重要因素，为研究化疗药物耐药性提供了重要的参考因素。

## Abstract (en)

**Background and objective:**

It has been proven that biomarkers play a key role in the individualized treatment of lung cancer. Correlations between expressions of TUBB3/STMN1 and gene mutations of epidermal growth factor receptor (*EGFR*), Kirsten rat sarcoma viral oncogene homolog (*KRAS*), v-raf murine sarcoma viral oncogene homolog B (*BRAF*), phosphatidylinositol-4, 5-bisphosphate 3-kinase (*PI3K*) in non-small cell lung cancer (NSCLC) would be investigated and this may provide essential significance on prognosis and predictions of drug efficiency.

**Methods:**

This study total enrolled 46 NSCLC patients. Tumor specimens were resected. The gene expressions of *TUBB3*, *STMN1* were analyzed using branched DNA-liquidchip, and *EGFR*, *KRAS*, *BRAF*, *PI3K* gene mutations were detected by xTAG-liquidchip. Correlations between gene expressions and gene mutations were further analyzed by Spearman.

**Results:**

Co-expressions between TUBB3 and STMN1 are strongly demonstrated, and high expression of TUBB3 always exists as well as high expression of STMN1 (*P*=0.006). Meanwhile mutation of *EGFR* E21 is negatively correlated with TUBB3, and wild type of *EGFR* E21 always exists toghther with high expression of TUBB3 (*P*=0.004, 6), whereas mutation of KRAS E2 is positively associated with expression level of STMN1 (*P*=0.038, 6).

**Conclusion:**

Antimicrotubule drug resistance factors of TUBB3 and STMN1 may be related with mutations of *EGFR* signal pathway, suggesting that *EGFR* mutation and *KRAS* mutation may be important factors in regulation of TUBB3/STMN1 expression, which provided basic references for chemotherapeutic resistance.

肺癌是当前世界上发病率和死亡率最高的恶性肿瘤，其中非小细胞肺癌（non-small cell lung cancer, NSCLC）的发病率最高，占80%左右^[1, 2]^。早期的NSCLC患者，治疗原则以手术为主，术后根据分期等进行辅助性化疗。然而在诸多辅助化疗方案中，仅有部分肿瘤患者从化疗获益。以铂类联合三代新药（长春新碱，紫杉醇、吉西他滨、培美曲塞等）是治疗NSCLC的基本方法，但有效率却仅达25%-30%^[3]^。可能与化疗反应率低或对联合化疗过程中产生的原发或继发性药物耐药有关。而肿瘤细胞检测相关基因表达水平的异常可能与肺癌产生耐药的有密切关系。最常见的化疗疗效预测因子是铂类药物耐药因子ERCC1和吉西他滨耐药因子RRM1^[4-6]^。抗微管因子TUBB3和STMN1是长春新碱、紫杉类、多西他赛等化疗药物的主要耐药因子，这些耐药因子与化疗药物的关系已经阐明^[7, 8]^。近期研究发现这些耐药因子受到肿瘤信号通路因子的调控，但还没有明确结论。本研究主要探讨抗微管类的靶点TUBB3和STMN1与表皮生长因子受体（epidermal growth factor receptor, EGFR）通路的关键因子的相关性。

## 1 资料与方法

### 1.1 病例选择

从53例NSCLC患者中筛选出完整临床病理资料的46例NSCLC手术组织标本（2012年5月-2013年5月）来自于中国人民解放军总医院胸外科，其中男性22例，女性24例，术后病理分期，T1期21例；T2期23例；T3期2例；T4期0例。

### 1.2 样本采集及检测方法

#### 1.2.1 样本处理

新鲜手术组织用10%中性福尔马林固定16 h-24 h，酒精洗涤固定后，送至广州益善医学检验所进行检测。

#### 1.2.2 mRNA表达检测技术

检测方法为分支DNA-液相芯片法，同时定量检测*TUBB3*、*STMN1*基因mRNA表达，分支DNA液相芯片技术是不进行RNA抽提、纯化和逆转录等步骤实现对mRNA表达水平的直接检测的技术。在裂解样本后，通过微球捕获，采用探针的多位点特异配对、级联放大的方式来实现信号的放大，读数时采用液相芯片进行结果的判读，可以在一次反应中同时检测多种靶标基因的mRNA表达水平，具体步骤包括：①取适量FFPE样本加入裂解液，56 ℃下裂解反应2 h；②预杂交：将样本裂解液转至孵育板上，加入支持探针-微球、支持延伸探针、缓冲液，55 ℃震荡孵育过夜；③吸取上清：次日将孵育板放在磁力架上1 min，此时磁性微球聚集在底部，吸取弃去上清；④洗涤：加入洗涤液，震荡洗涤1 min，孵育板放在磁力架上1 min，吸取弃去上清；重复3次；⑤杂交：加入扩增延伸探针和标记探针，50 ℃震荡反应1 h；⑥洗涤：孵育板放在磁力架上1 min，吸取弃去上清；用洗涤液洗2次；⑦信号放大：加入链霉亲和素-藻红蛋白，50 ℃震荡反应30 min；⑧洗涤：孵育板放在磁力架上1 min，吸取弃去上清；用洗涤液洗2次；⑨读数：加入洗涤液，震荡5 min，于Luminex阅读仪上读取数据；⑩分析：数据分析，得出检测结果。

#### 1.2.3 基因突变检测技术

采用SurPle^®^x-xTAG70plex液相芯片技术对组织样品进行*EGFR*、*KRAS*、*BRAF*、*PI3K*基因突变检测。该技术建立在Luminex平台上，是一种集合流式细胞技术、激光技术、数字信号处理技术及传统芯片技术为一体的新型生物检测技术，液相芯片技术的技术步骤可分为微球编码、探针偶联、液相悬浮反应和检测分析。通过多重PCR方法获得各基因外显子上含有常见等位基因型的基因片段，进行等位基因特异引物延伸（allele specific primer extension, ASPE）反应，ASPE引物上的Tag（微球）序列与聚苯乙烯微球上的Anti-Tag序列特异结合，完成杂交反应，杂交后的微球通过Luminex 200系统进行分析，读取每个样品的中位荧光值（MFI）。

### 1.3 统计学方法

应用SPSS 15.0软件进行统计分析。采用*Shapiro-Wilk*（简称*W*检验）、*Kolmogorov-Smirnov*检验（检测*K*S检验）与*D*检验，进行正态分布的考察。根据正态分布结果，选择*Pearson*或*Spearman*相关性分析。以*P*=0.05为检验水准，以*P* < 0.05为差异有统计学意义。

## 2 结果

### 2.1 患者的临床基线特征

46例NSCLC患者临床特征描述见表 1。

**1:** 患者基线临床特征描述 Characteristics of patients

Characteristic	No. (*n*=46, %)
Age	54.36 (mean)
Median (range), years	52.50 (median); 35-78 (range)
Gender	
Male	22 (47.83%)
Female	24 (52.17%)
Histological	
Adeno	39 (84.78%)
Squamous	5 (10.87%)
Other	2 (4.34%)
Differentiated	
Poorly	8 (18.18%)
Moderately-Poorly	7 (15.91%)
Moderately	20 (45.45%)
Well-Moderately	5 (11.36%)
Well	4 (9.09%)
Missing	2
Stage	
Ⅰa	16 (34.78%)
Ⅰb	13 (28.26%)
Ⅱa	1 (2.17%)
Ⅱb	1 (2.17%)
Ⅲa	12 (26.09%)
Ⅲb	1 (2.17%)
Ⅳ	2 (4.35%)
Missing	0
Tumor size	
T1	21 (45.65%)
T2	23 (50.00%)
T3	2 (4.35%)
T4	0 (0.00%)
Missing	0
Clinical nodal status	
N0	30 (65.22%)
N1	5 (10.87%)
N2	10 (21.74%)
N3	1 (2.17%)
Missing	0
Metastasis	
Negative	44 (95.65%)
Positive	2 (4.35%)
Missing	0

### 2.2 TUBB3及STMN1 mRNA表达水平检测

采用分支-DNA液相芯片技术对TUBB3及STMN1进行检测，内参基因为一组基因（*B2M*, *TBP*, *TFRC*）。不同于Q-PCR，该技术无需RNA提取及PCR扩增。46例NSCLC标本中TUBB3的平均表达水平为0.185（0.07-0.721），STMN1的平均表达水平为1.328（0.70-4.80）。

### 2.3 EGFR通路关键基因突变检测

采用xTAG技术进行*EGFR* E18、E19、E20、E21，*KRAS* E2、E3，*BRAF*，*PI3K* E9、E20突变分析。结果如表 2所示，46例标本中，*EGF*R突变率50%（23/46），*KRAS*突变率13.1%（6/46）。*PI3K*突变率2.2%（1/46），没有发现*BRAF*突变。

**2:** 基因突变分析 Mutations of genes

Gene	Wild Type	Mutated
*EGFR*_E18	97.8% (45)	2.2% (1)
*EGFR*_E19	76.1% (35)	23.9% (11)
*EGFR*_E20	100% (46)	-
*EGFR*_E21	76.1% (35)	23.9% (11)
*KRAS*_E2	89.1% (41)	10.9% (5)
*KRAS*_E3	97.8% (45)	2.2% (1)
*BRAF*	100% (46)	-
*PIK3CA*	97.8% (45)	2.2% (1)

### 2.4 正态分布检验及相关性分析

正态性是进行*Pearson*关联性分析的前提假设，若变量不满足正态性，则不能采用*Pearson*相关进行分析，只能采用*Spearman*相关进行分析。因此在进行相关性分析前应对变量的正态性进行检验。本研究采用*Shapiro-Wilk*（简称*W*检验）、*Kolmogorov-Smirnov*检验（检测*KS*检验）与*D*检验考察TUBB3和STMN1的正态分布，如果*P*值大于0.10可认为变量服从正态分布。本研究的STMN1和TUBB3的所有正态性检验结果都不满足*P*大于0.10这个条件，可认为STMN1与TUBB3不服从正态分布，因此，本研究不能采用*Pearson*相关进行分析，而采用*Spearman*相关进行分析。

### 2.5 TUBB3/STMN1及EGFR通路临床病理特征与临床特征的相关性

结果显示，TUBB3及STMN1的mRNA水平的表达与性别、年龄、分化程度、远处转移等病理特征没有明显差异。*EGFR* E19突变与吸烟史相关（*P*=0.001, 2），与其他病理特征没有明显差异（表 3）。

**3:** 基因表达及突变与临床病理特征的相关性。 Correlation between gene and pathology characteristics

		*TUBB3*	*STMN1*	*EGFR* E18	*EGFR* E19	*EGFR* E21	*KRAS* E2
Gender	*χ*^2^	0.201, 27	-0.248, 63	0.207, 2	0.119, 2	0.111, 9	0.164, 74
	*P*	0.190, 2	0.103, 7	0.188, 3	0.452, 1	0.480, 5	0.297, 2
Age	*χ*^2^	0.204, 65	-0.385, 91	0.263, 75	-0.121, 14	0.151, 28	0.218, 86
	*P*	0.182, 7	0.700, 0	0.091, 5	0.444, 7	0.338, 9	0.163, 8
Stage	*χ*^2^	0.216, 07	-0.414, 09	0.396, 16	-0.161, 33	0.290, 01	0.195, 53
	*P*	0.158, 9	0.102, 0	0.094, 0	0.307, 4	0.062, 5	0.214, 6
Distant metastasis	*χ*^2^	0.360, 54	-0.011, 13	0.290, 82	-0.028, 44	-0.043, 51	0.472, 17
	*P*	0.076, 2	0.942, 8	0.061, 7	0.858, 1	0.784, 4	0. 162, 0
Differentiation	*χ*^2^	0.187, 74	-0.130, 66	0.0512, 9	0.065, 88	-0.181, 91	0.197, 8
	*P*	0.222, 3	0.397, 9	0.747, 0	0.678, 5	0.248, 9	0.209, 3
Smoking history	*χ*^2^	0.137, 42	0.448, 06	0.254, 19	0.745, 61	0.177, 22	0.315, 78
	*P*	0.373, 7	0.062, 3	0.104, 3	0.001, 2	0.069, 2	0.071, 6

### 2.6 TUBB3/STMN1与EGFR通路关键因子突变相关性分析

采用*Spearman*方法分析TUBB3/STMN1 mRNA表达之间的相互关系，结果显示：TUBB3和STMN1存在很强的共表达性，*TUBB3*基因高表达时，STMN1趋向于高表达（*P*=0.006）。通过分析TUBB3/STMN1 mRNA与*EGFR*突变及*KRAS*突变的相互关系，结果显示：*EGFR* E21突变与TUBB3存在负相关关系：*EGFR* E21无突变时TUBB3趋向于高表达（*P*=0.004, 6）。*KRAS* E2突变与STMN1存在正相关关系：*KRAS* E2突变时STMN1趋向于高表达（*P*=0.038, 6）（表 4）。

**4:** 基因相关性分析 Correlations between genes

Gene（*P* value）	*TUBB3*	*STMN1*
*EGFR* E18	0.138, 11	-0.222, 17
	0.371, 3	0.147, 2
*EGFR* E19	0.018, 6	0.179, 8
	0.904, 6	0.242, 9
*EGFR* E21	-0.419, 53	-0.101, 27
	0.004, 6	0.513, 1
*KRAS* E2	0.172	0.312, 99
	0.264, 2	0.038, 6
*TUBB3*	1	0.408, 03
		0.006
*STMN1*	0.408, 03	1
	0.006	

在多重检验时需将检验水准进行调整，结果才具有更高的重复性。本文中*P*值计算经*Bonferroni*法调整后，检验水准设定为0.0004。

## 3 讨论

随着人类基因组学及功能基因组学研究的不断深入，分子标志物指导肺癌个体化治疗已成趋势。根据患者基因状况等个体差异来制订针对性的治疗方案，可以避免不当治疗及无效治疗，而确定个体差异则主要通过检测分析患者特定基因的表达水平或突变来实现。

多项研究发现切除修复交叉互补基因1（ERCC1）、核糖核苷酸还原酶M1（RRM1）、微管蛋白β-Ⅲ（TUBB3）、Stathmin 1（STMN1）、胸苷酸合成酶（TYMS）、与NSCLC的化疗疗效直接相关^[7-10]^。本研究主要针对抗微管类的耐药因子TUBB3及STMN1进行。

细胞内微管蛋白分α、β两个亚型，是有丝分裂时纺锤体的基本组成单位，也是抗微管药物的主要作用靶点。研究显示，TUBB3编码的Tubulin-Ⅲ（β-3型微管蛋白）与抗微管化疗药敏感性的关系最密切。高TUBB3表达水平与长春瑞滨的耐药性和生存期较短相关^[11, 12]^。

*STMN1*基因同样是抗微管类药物的靶点，该基因编码的STMN1蛋白通过促进微管的解聚或阻止微管的聚合从而影响有丝分裂纺锤体的形成^[12]^。研究表明*STMN1*基因的mRNA表达水平与抗微管类化疗的疗效密切相关。其中一项NSCLC的临床研究显示，STMN1高表达患者紫杉类化疗有效率低28.33%，而STMN1低表达患者化疗有效率高60.00%（*P*=0.021）^[13]^。

肿瘤细胞EGFR信号通路上基因的突变状态决定着EGFR靶向药的疗效，研究得出与其疗效有关的突变位点主要有*EGFR*外显子18、19和21的突变，*KRAS*外显子2和3的突变，*BRAF* V600E点突变以及*PI3K*外显子9和20的突变^[14, 15]^。*KRAS*的突变在NSCLC患者中的发生率为10%-20%。既往研究证实*KRAS*的突变与性别、年龄、肿瘤分期以及临床表现都没有明显联系，但在不吸烟患者和病理类型为腺癌的患者中发生的几率明显升高。另有研究表明*KRAS*突变会影响EGFR-TKIs治疗的疗效，对存在*KRAS*基因突变的NSCLC患者应用EGFR-TKIs治疗的疗效明显降低，*KRAS*基因突变状态可以作为NSCLC患者是否采用EGFR-TKIs治疗的筛选标准^[15, 16]^。

本研究主要分析*EGFR*突变、*KRAS*突变与TUBB3及STMN1的mRNA表达之间的关系，探索不同分子标志物的相关性，尝试揭示化疗药物的耐药性机制。2008年的IPASS研究是肺癌个体化治疗的里程碑，该研究除了发现*EGFR*突变与EGFR-TKI疗效明确相关外，也发现了*EGFR*敏感突变的患者接受化疗的效果要比*EGFR*野生型患者效果好^[17]^，提示了*EGFR*突变可能与化疗的耐药因子的相关性。随后的相关研究发现：*ERCC1*基因与*EGFR*突变存在相关性，*EGFR*突变时，ERCC1趋向于低表达^[18]^。

本研究中分析了TUBB3和STMN1和EGFR信号通路的关系。检测mRNA水平采用的方法是分支-DNA液相芯片，检测过程中无需进行RNA抽提、纯化和逆转录等，一方面检测结果基本不受样品中RNA降解等因素的影响，保证了检测结果的准确性；另一方面，液相芯片以多个持家基因作对照（*n*≥3），准确性高使检测结果更为可靠。同时，本液相芯片使用的是探针的多位点特异配对、级联放大的方式来实现信号的放大，与传统的real time PCR（qPCR）方法相比，可同时检测超过30个基因，大大减低操作过程或多步骤误差积累对结果的影响。

本研究检测基因突变的方法是xTAG液相芯片方法，该方法可同时检测EGFR通路70个位点，真正做到多靶标联合检测。与其他技术相比，该技术具有灵敏高、特异性强、量化可重复、操作简便、结果判定标准化等突出优势，实现了高度灵敏、重复性好的多指标基因突变检测在临床肿瘤靶向治疗的运用。

在选择可靠技术的前提下，我们对结果进行了分析。通过分析基因与临床病理特征的相关性发现，由于本研究中样本量较少，没有发现基因与临床病理特征如性别、年龄、肿瘤大小等的明显差异。由于随访数据还在进行，本文无法分析TUBB3及STMN1与预后的相关性。

针对EGFR通路的研究中，本文发现EGFR敏感性突变（*EGFR* E18、E19、E21）的突变率达50%，*KRAS*突变率13.1%。文献中报道的中国人群EGFR突变率一般30%-40%，这可能与本文的样本量较少有关。在结果中发现不吸烟的患者中更容易出现*EGFR* E19的突变，这与之前的报道较为一致，而*KRAS*的突变和吸烟史没有发现显著性差异。

关于TUBB3/STMN1与*EGFR*突变的相关性的报道较少。Levallet等2012年报道显示：*KRAS*突变导致TUBB3高表达（*P* < 0.001）的主要因素，通过基因敲除实验，证明了KRAS信号通路可能是调控*TUBB3*基因表达的关键因子^[19]^，提示了基因突变在化疗药物耐药的机制的作用。

本研究的*TUBB3*和*STMN1*基因表达的相关性分析结果显示：TUBB3和STMN1存在共表达。将TUBB3和STMN1共同与EGFR通路关键因子突变进行相关性分析，结果发现：*EGFR* E21无突变时TUBB3趋向于高表达（*P*=0.004, 6），而STMN1表达与*EGFR* E21有相关性，但没有发现显著差异（*P*=0.513, 1）。*KRA*S E2突变与STMN1存在正相关关系，*KRAS* E2突变时STMN1趋向于高表达（*P*=0.038, 6）。而TUBB3表达与*KRAS*突变有相关性，但没有显著性差异（*P*=0.26）。

这些结果显示EGFR信号通路的关键因子EGFR、KRAS在调控*TUBB3*/*STMN1*基因表达方面可能起着关键作用。TUBB3/STMN1都是抗微管药物的相关靶点，抗微管类药物是作用于细胞微管，通过影响纺锤体的形成，从而抑制细胞有丝分裂。STMN1/TUBB3的表达水平与有丝分裂的活动相关，抑制这些基因的表达，可以干扰细胞的各种功能，从而影响肿瘤细胞的增殖与凋亡。本文研究结果发现，*KRAS*的突变会导致STMN1的高表达，表明了EGFR通路中的基因活化状态与细胞的增殖活动密切相关。Levallet报道中称，*TUBB3*基因在支气管细胞中的表达与PI3K/AKT/mTOR通路或KRAS/RAF/MEK等通路相关，这些通路上基因的活化状态，是否影响着细胞的有些分裂，需要进一步的RNAi试验及PI3K或MEK抑制剂来研究，确定下一步的机制，从而为化疗耐药机制提供重要基础。

本研究没有显示出两个指标与*EGFR*突变或*KRAS*突变的一致相关性。这些可能是由于样本数量少的原因，需要扩大样本量进一步验证。也可能EGFR通路在调控两个基因的表达时，存在差异性。这些都需要在进一步的大样本研究中验证。由于*BRAF*、*PI3K*基因在本文中突变较少，达不到统计学要求，因此没有进行相关性分析，这些因子是否也调控着TUBB3/STMN1的表达，需要进一步的研究来验证。

本研究初步分析了TUBB3/STMN1表达与EGFR信号通路的关键因子*EGFR*、*KRAS*突变的关系，初步结果验证了该信号通路在调控化疗耐药基因表达的作用。为个体化分子标志物提供了很好的依据。提示了个体化分子标志物临床应用过程中，联合多个分子标志物的分析，能提供更系统的参考依据，而接下来的工作需要进一步研究调控的机制。
